# Utility of Botulinum Injections in Stiff-Person Syndrome

**DOI:** 10.1155/2019/9317916

**Published:** 2019-10-07

**Authors:** L. M. Conners, A. Betcher, A. Shahinian, P. Janda

**Affiliations:** Department of Neurology, Valley Hospital Medical Center, Las Vegas, NV, USA

## Abstract

Stiff-person syndrome (SPS) is an uncommon neurological disorder characterized by significant rigidity and muscle spasms primarily affecting the truncal and proximal musculature. Furthermore, a wide-based gait with functional impairment is generally seen. High-dose benzodiazepines or baclofen are widely considered the optimal initial therapy; however, major adverse effects often preclude adequate dosing. Refractory cases may be treated with intravenous immunoglobulins (IVIG), plasma exchange, or B-cell depletion with rituximab, although these are also associated with major, sometimes fatal, adverse reactions. Several reports have validated the safety and utility of botulinum injections in this setting, yet botulinum remains markedly underutilized in this cohort. Below, a case report and review of the literature show botulinum can decrease pain and stiffness, improve gait and balance, and decrease dependence on powerful systemic treatments in this group.

## 1. Introduction

The first reports of stiff-person syndrome (formerly stiff-man syndrome) were published by Moersch and Woltman in 1956, and described “tin soldiers” that were “board-like” and “stiff” [1]. This stiffness results from dysfunction of CNS inhibitory pathways involving glutamic acid decarboxylase (GAD) and gamma amino butyric acid (GABA) in these patients. GAD is an enzyme responsible for the conversion of L-glutaminc acid to GABA and is critical in maintaining inhibitory pathways [2]. Sixty to eighty percent of those with SPS have elevated titers of antibodies against GAD-65 in their serum [3].

Epidemiologic data are somewhat limited due to underdiagnosis of the disease, but the current estimated prevalence of SPS is approximately one per million. Patients typically present between the ages of twenty and fifty. As with many autoimmune phenomena, there is a female predominance, 2 : 1. There are associations with other autoimmune processes, particularly type 1 diabetes mellitus [4].

Many describe stiff-person syndrome as a spectrum, with more than eighty percent of patients falling into the category of classic SPS [5]. Variants of SPS described in the literature include partial stiff-person syndrome, paraneoplastic stiff-person syndrome, and progressive encephalomyelitis with rigidity and myoclonus (PERM). Partial SPS, also known as stiff-limb syndrome, is the most common variant and affects ten to fifteen percent of patients on the SPS spectrum. While classic SPS is characterized by truncal and proximal spasms and rigidity, stiff-limb syndrome is typically associated with focal rigidity of a lower limb, sparing the trunk muscles. These patients typically lack auto-antibodies to GAD and respond less adequately to benzodiazepines and baclofen [6]. Functionally, they suffer significant gait disturbances due to considerable rigidity of the lower limb, and many are unable to ambulate altogether [3, 6]. Alternatively, patients with the paraneoplastic variant of SPS share clinical features of the classic stiff-person syndrome and may present with a known diagnosis of neoplasm or concomitant features of malignancy, such as unintentional weight loss. They are typically GAD seronegative. Accordingly, this diagnosis should always be considered in those with strong features of classic stiff-person syndrome who lack auto-antibodies to GAD in the serum. Paraneoplastic SPS has been associated with breast cancer, lung cancer, and Hodgkin's lymphoma, and is estimated to affect one to two percent of patients with SPS overall [3]. Finally, less than one-tenth of one percent of patients will be diagnosed with progressive encephalomyelitis with rigidity and myoclonus, which includes transient oculomotor disturbances not seen in classic SPS or other variants. Features of classic stiff-person syndrome with brainstem dysfunction, myoclonus, sensory deficits, sphincter, or autonomic dysfunction, seizures, or cognitive deficits may suggest a diagnosis of progressive encephalomyelitis with rigidity and myoclonus [3, 5]. It typically takes a relentless course, often leading to death within a few months [6].

There are no formally accepted diagnostic criteria for stiff-person syndrome, and establishing the diagnosis requires a high level of suspicion on the part of the examiner. Stiffness in the axial or limb muscles resulting in an impaired gait, in combination with episodic spasms (often provoked by sudden movement, noise or emotional upset) are generally required to make the diagnosis. Improvement with GABA-enhancing medications such as diazepam and continuous motor unit activity on EMG are expected as well. Symptoms should not be better explained by another condition. The presence of anti-GAD antibodies is not required but supports the diagnosis when present [3].

Current therapies aim to mitigate painful spasms and improve mobility. Initial therapy typically consists of diazepam, or clonazepam when diazepam is poorly tolerated. Very high dosages are usually required to adequately control symptoms, with up to thirty milligrams four times a day being a typical dose of diazepam for these patients. Benzodiazepines, however, are oftentimes limited by major adverse effects including somnolence, fatigue, mood changes, or respiratory depression. If benzodiazepines fail to adequately control symptoms or are poorly tolerated, baclofen is usually used. When baclofen is used in combination with benzodiazepines, sedation may be significant and limit adequate dosing. Benzodiazepines and baclofen improve symptoms by augmenting the inadequate GABA-ergic inhibitory pathways in these patients [3]. For those with refractory symptoms or who are intolerant of the above therapies, IVIG has been shown to improve mobility and unassisted ambulation [7]. B-cell depletion with rituximab has also been used in this cohort. Notably, IVIG and rituximab should be reserved for refractory cases given the potential for serious adverse effects. Other, therapies may include botulinum, plasma exchange, intravenous methocarbamol, sodium valproate, gabapentin, vigabatrin, and propofol [4, 8–12].

SPS is a progressive disease. Initiation of therapy is recommended as soon as a diagnosis is made as the natural course of the disease involves progressive gait dysfunction and limitations in functioning. Combination therapies are usually recommended to maximize functional status [3].

## 2. Discussion

Our discussion begins with a case report describing the use of botulinum injections in a patient with classic stiff-person syndrome and concludes with a review of the literature on this topic. A 38-year-old female with anxiety, depression, and Grave's disease presented with several months of diffuse muscle pain and spasms. She had previously been diagnosed with hyperthyroid myopathy as well as rhabdomyolysis. Repeat CK level in the clinic was normal, as was MRI of the brain and cervical spine, EMG/nerve conduction study, aldolase, autoimmune panel, and muscle biopsy. Notably, anti-GAD antibodies were negative in the serum at that time. One year later, she returned with a progressive worsening of her symptoms, including impaired ambulation, difficulties with activities of daily living, and hospitalizations to manage pain. Repeat serum anti-GAD-65 antibodies were 69 IU/mL (normal <5 IU/mL). EMG revealed continuous motor activity in several muscles of the neck and thoracic paraspinal muscles, where she reported significant pain and tightness. Her symptoms were not sufficiently controlled on a combination of diazepam 7.5 mg three times a day, baclofen 30 mg three times a day, Percocet 10–325 mg four times per day, tizanidine 4 mg three times a day, IVIG 400 mg/kg every 4 weeks, and physical therapy. Her initial Botox treatment consisted of 25 units Botox in the right as well as left trapezius, and 25 units in the right and left sternocleidomastoid muscle. Three months later she had a second treatment in which those dosages were exactly doubled (50 units in the right and left trapezius and 50 units in the left and right sternocleidomastoid muscles). Again, three months afterwards she received 50 units in the right and left trapezius and sternocleidomastoid muscles as well as 20 units in the right as well as left thoracic paraspinals as she noted pain and spasms in that region. 6 months later she received 40 units in the right and left trapezius, 40 units in the right and left levator scapulae, and 30 units in the right and left semispinalis capitis muscles. The Botox dosages were adjusted each treatment to target the most painful regions at that time. After the addition of Botox injections, the patient reported an improvement in pain, decreased muscle spasms, an improvement in her quality of life, although she was not able to decrease her home medications. She denied adverse effects from the Botox treatments.

In 2003, Szczepańska-Szerej et al. reported a marked functional improvement and a decreased dependence on systemic drugs for up to four months in a 41-year-old woman with SPS following botulinum injections [13]. Similarly, Shah and Bunzol described a 53-year-old-male with stiff-person syndrome with a poor response to IVIG, corticosteroids, diazepam, baclofen, gabapentin, warm baths, heating pads, transcutaneous electrical nerve stimulation, and chiropractic manipulation. Following botulinum injections to the biceps femoris, adductor magnus, and rectus femoris, the patient reported considerable pain relief, improved range of motion, and decreased stiffness. He also noted an improvement in balance and gait with fewer falls and the ability to walk longer distances [14]. Likewise, in 1993, Davis and Jabbari reported remarkable relief from pain and stiffness and a substantial improvement in ambulation in a 36-year-old male with stiff-person syndrome after botulinum injections into the lumbar paraspinal muscles. The patient suffered from an awkward and antalgic gait and was unable to stand from the sitting position without assistance due to marked stiffness and pain. EMG noted continuous activity in the lumbar paraspinals, which were injected with a total of 560 units of botulinum toxin after diazepam and baclofen provided only minimal relief. The injections were done over three sessions spanning three weeks and were divided over five sites from L1–L5. One week after the final set of injections, the patient noted almost complete relief from exertional pain and resolution of painful spasms. The injections also lead to a significant functional improvement and reduced need for systemic drugs [2].

Botulinum injections have also been shown to improve rigidity, pain, and gait in stiff-limb syndrome. In 2012, Anagnostou and Zambelis recounted a 40-year-old female with several years of progressive stiffness of the left leg. Modified Ashworth scale score of the extremity was four, signifying that the affected extremity was rigid in flexion or extension, and EMG showed continuous motor unit potentials in the left vastus medialis. Following a poor response to pharmacotherapy, several dosages of Botox were tried over time without significant subjective improvement. Ultimately, a dose of 900 units (vastus lateralis 350 units, vastus medialis 350 units, and rectus femoris 200 units) produced a marked improvement in her symptoms. She reported a dramatic improvement in rigidity and gait as well as decreased pain for two and a half months following the injections. Modified Ashworth scale score following the injections was one, denoting a slight increase in muscle tone, and repeat EMG was within normal limits. She denied adverse effects other than a slight weakness of leg extension, which she noticed only when attempting to jump. Three months following the injections, her symptoms had returned. She repeated the injections at the dose of 900 units and again reported a dramatic improvement in her symptoms [15].

## 3. Conclusion

SPS is a progressive neurological condition that often leads to refractory rigidity, painful spasms, and gait difficulties. The use of botulinum toxin in this cohort has been shown to improve function, gait, and quality of life, decrease pain and spasms, and decrease the need for systemic drugs; however, its use continues to remain markedly underutilized in this group.
